# Psychiatric sequelae of childhood sexual abuse: a case series of six hospitalized minors in Moldova

**DOI:** 10.1192/j.eurpsy.2026.11124

**Published:** 2026-07-31

**Authors:** L. Sanduleac

**Affiliations:** Mental Health, Psychotherapy and Medical Psychology, State University of Medicine and Pharmacy “Nicolae Testemiţanu”, Chisinau, Moldova, Republic of

## Abstract

**Introduction:**

Childhood sexual abuse (CSA), a severe form of adverse childhood experience (ACE), is strongly associated with enduring psychiatric morbidity. This study describes the clinical profiles, diagnoses, and early treatment outcomes of six minors hospitalized in 2025 in a psychiatric facility following documented sexual abuse.

**Objectives:**

The study aimed to document the clinical characteristics of minors hospitalized after childhood sexual abuse, with particular focus on their psychiatric diagnoses, symptom profiles, and short-term treatment outcomes. In addition, it sought to identify underlying psychosocial and familial risk factors influencing vulnerability and prognosis, while underscoring the importance of trauma-informed, multidisciplinary approaches to psychiatric care in this population.

**Methods:**

A retrospective review of medical records was conducted for six patients aged 5–15 years (4 females, 2 males). Collected data included socio-demographics, family and psychiatric history, nature of abuse, symptomatology at admission, diagnoses, treatment regimens, and short-term clinical course.

**Results:**

All patients disclosed severe CSA, either intrafamilial (primarily paternal perpetrators) or extrafamilial. Contributing psychosocial risk factors included parental neglect and alcohol dependence. Presenting symptoms encompassed affective dysregulation, sleep impairment, irritability, somatic complaints, and social withdrawal. Two minors expressed suicidal ideation, while one developed early-onset schizophrenia with psychotic manifestations. Diagnoses ranged from conduct and emotional disorders (ICD-10 F92.8) to major depressive disorder, recurrent, severe with psychotic features (F33.3), and undifferentiated schizophrenia (F20.3). Management included antipsychotics (Risperidone, Aripiprazole), mood stabilizers (Carbamazepine), and supportive care. Prognosis remained guarded, with recurrent hospitalizations in the most severe cases.

**Conclusions:**

These cases highlight the profound psychiatric burden of CSA in minors, spanning emotional, affective, and psychotic disorders. The emergence of suicidal ideation in early adolescence underscores the acute risk for self-harm, while the occurrence of early-onset schizophrenia illustrates the possible triggering role of trauma in severe mental illness. Findings emphasize the need for systematic trauma-informed screening, specialized interventions, and integrated multidisciplinary psychiatric care for abused children.

**Disclosure of Interest:**

None Declared

